# Calprotectin — A Novel Marker of Obesity

**DOI:** 10.1371/journal.pone.0007419

**Published:** 2009-10-12

**Authors:** Ole Hartvig Mortensen, Anders Rinnov Nielsen, Christian Erikstrup, Peter Plomgaard, Christian Philip Fischer, Rikke Krogh-Madsen, Birgitte Lindegaard, Anne Marie Petersen, Sarah Taudorf, Bente Klarlund Pedersen

**Affiliations:** The Centre of Inflammation and Metabolism, Department of Infectious Diseases and Copenhagen Muscle Research Centre, Rigshospitalet, Faculty of Health Sciences, University of Copenhagen, Copenhagen, Denmark; Mayo Clinic College of Medicine, United States of America

## Abstract

**Background:**

The two inflammatory molecules, S100A8 and S100A9, form a heterodimer, calprotectin. Plasma calprotectin levels are elevated in various inflammatory disorders. We hypothesized that plasma calprotectin levels would be increased in subjects with low-grade systemic inflammation i.e. either obese subjects or subjects with type 2 diabetes.

**Methodology/Principal Findings:**

Plasma calprotectin and skeletal muscle S100A8 mRNA levels were measured in a cohort consisting of 199 subjects divided into four groups depending on presence or absence of type 2 diabetes (T2D), and presence or absence of obesity. There was a significant interaction between obesity and T2D (p = 0.012). Plasma calprotectin was increased in obese relative to non-obese controls (p<0.0001), whereas it did not differ between obese and non-obese patients with T2D (p = 0.62). S100A8 mRNA levels in skeletal muscle were not influenced by obesity or T2D. Multivariate regression analysis (adjusting for age, sex, smoking and HOMA2-IR) showed plasma calprotectin to be strongly associated with BMI, even when further adjusted for fitness, CRP, TNF-α or neutrophil number.

**Conclusions/Significance:**

Plasma calprotectin is a marker of obesity in individuals without type 2 diabetes.

## Introduction

Recently, two novel inflammatory molecules belonging to the S100 protein family have been described, S100A8 (MRP8) and S100A9 (MRP14)[1]. S100A8 and S100A9 form monovalent homodimers and a heterodimer, known as calprotectin, in a calcium-dependent manner. Both proteins are predominantly expressed in myeloid cells depending on the stage of cell differentiation and inflammatory status[1], however they have also been found to be expressed in skeletal muscle[2]. Studies on the regulation of S100A8 and S100A9 have shown that S100A9 seems to be ubiquitously expressed, while S100A8 can be induced by a variety of stimulants such as lipopolysaccharide, interferon-γ and tumor necrosis factor-α[3]. S100A8 and S100A9 take part in various processes of the innate immune response such as cell adhesion, chemotaxis, and antimicrobial activity[1]. Phagocytes expressing S100A8 and S100A9 and elevated plasma calprotectin levels are found in a variety of chronic inflammatory conditions, including rheumatoid arthritis, allograft rejections, and inflammatory bowel and lung diseases[4]. Calprotectin has also been found to be increased in plasma following exercise[5] and was recently shown to be released from skeletal muscle tissue [6].

We hypothesized that plasma calprotectin and skeletal muscle expression of S100A8 would be dysregulated in individuals with systemic inflammation. As low-grade systemic inflammation is a hallmark of both obesity and type 2 diabetes (T2D) we studied the levels of plasma calprotectin and S100A8 skeletal muscle mRNA levels using a cross-sectional case control design in which patients with T2D and healthy controls were closely matched, not only according to age and sex but also according to body mass index (BMI).

## Materials and Methods

### Cohort study

#### Ethics Statement

The participants received oral and written information about the experimental procedures before giving their written informed consent. The study was approved by the Ethical Committee of Copenhagen and Frederiksberg Communities, Denmark (KF 01-141/04), and performed according to the Declaration of Helsinki.

### Subjects

A cross-sectional design was employed. Participants (n  =  199) were divided into 4 groups according to BMI (above or below 30 kg/m2) and diagnosis of T2D. Subjects and protocol have previously been described[7], [8].

### Protocol

Participants reported in the laboratory between 8 and 10 am after an overnight fast. They did not take any medication in the 24 h preceding the examination and those with T2D did not take oral antidiabetic medication for one week prior to examination day. A general health examination was performed; blood samples were drawn from an antecubital vein, a skeletal muscle biopsy was obtained, an oral glucose tolerance test (OGTT), a fitness test was performed, and subjects were scanned on a dual-energy X-ray absorptiometry whole body scanner as previously described[7], [8].

### Blood analysis

Plasma calprotectin was measured using a commercial Enzyme Linked Immunosorbent Assay (ELISA) kit (Hycult biotechnology, Uden, NL). All measurements were performed in duplicate. Analysis of other plasma parameters has been previously described[7], [8].

### Skeletal muscle tissue S100A8 mRNA

Skeletal muscle tissue biopsies were obtained from musculus quadriceps and RNA isolated as previously described[8]. Following cDNA synthesis (Applied Biosystems, Foster City, CA, USA), real-time PCR was performed on an ABI 7900 Sequence Detection System (Applied Biosystems) using pre-developed TaqMan assays (Applied Biosystems) for S100A8 (Hs00374263_m1) and the endogenous control, β-actin. The mRNA content of both targets and the endogenous control, β-actin, was calculated from the cycle threshold values using the standard curve method and relative expression of S100A8 determined after normalisation to β-actin.

### Statistics

All analyses were performed with SAS 9.1.2 (SAS Institute, Cary, NC, USA. Plasma levels of triglycerides (TAG), glucose, insulin, HOMA2-IR, HbA1c, IL-6, TNF-α, c-reactive protein (CRP), plasma calprotectin, fitness (expressed as VO_2max_ pr. kg fat free mass), and S100A8 mRNA levels were log10-transformed to approximate normal distribution. For these parameters results are presented as geometric means with 95% confidence intervals (CIs). For all other parameters results are presented as means with CIs. Differences between glycaemia and obesity groups were tested in a two-way analysis of variance (ANOVA) (PROC GLM with the LSMEANS statement using Tukey HSD as post-hoc test). Univariate and multivariate regression analysis (PROC REG) was performed with plasma calprotectin as the dependent variable and parameters of obesity, inflammation, metabolism, and T2D as explanatory variables. Results are presented as regression coefficients with CIs. P<0.05 was considered significant.

## Results

### Plasma calprotectin is increased in obese subjects

**Figure 1:**
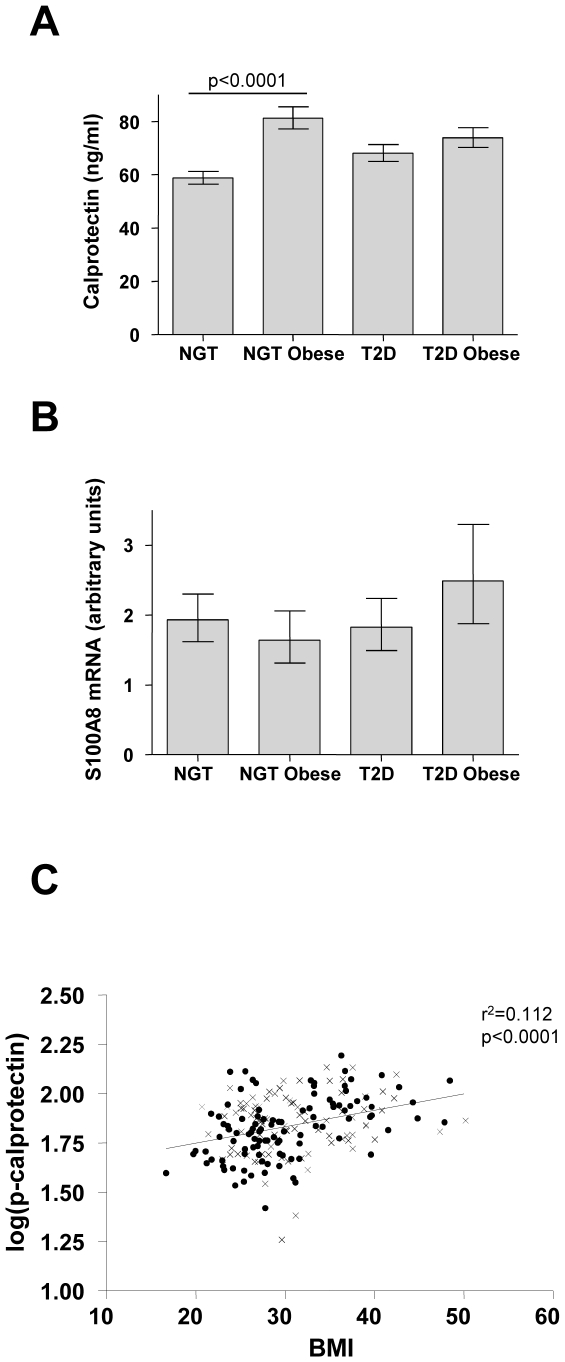
Plasma calprotectin and skeletal muscle mRNA levels in humans. A) Plasma concentrations of calprotectin are shown for the following four groups: Normal glucose tolerance (NGT)/Non-obese (N = 62), NGT/obese (N = 41), type 2 diabetes (T2D)/non-obese (N = 50), T2D/obese (N = 46). Data are expressed as geometric means; error bars represent 95% confidence intervals (CIs). B) Skeletal muscle S100A8 mRNA are shown for the following four groups: NGT/Non-obese (N = 53), NGT/obese (N = 37), T2D/non-obese (N = 44), T2D/obese (N = 31). Data are expressed as geometric means; error bars represent CIs. C) Log-transformed plasma concentrations of calprotectin, log(p-calprotectin), and BMI, with circles and crosses depicting NGT and T2D subjects, respectively. There was a significant correlation between log(p-calprotectin) and BMI (r^2^ = 0.112, p<0.0001).

General characteristics of the study population are shown in Table 1. For plasma calprotectin, an interaction was found between glycemia group and obesity in a two-way ANOVA (p = 0.012). Post-hoc tests showed that plasma calprotectin was increased in obese relative to non-obese controls (p<0.0001), whereas plasma calprotectin did not differ between obese and non-obese patients with T2D (p = 0.62) (Fig. 1A). The difference in plasma calprotectin between obese and non-obese controls persisted after adjusting for age and sex. Skeletal muscle S100A8 mRNA levels did not differ between groups (Fig. 1B).

**Table 1:** General characteristics of the study population and plasma calprotectin univariate and multivariate regression analysis.

	Subject characteristics												Regression analyses				
	Normal Glucose Tolerance					Type 2 Diabetes							Univariate		Multivariate		
	Non-obese		Obese			Non-obese		Obese					RC	p-value	RC	95% CI	p-value
n (male/female)	62	(42/20)	41	(28/13)		50	(38/12)	46	(34/12)				–	–	–	–	–
Age (years)	56	(53–59)	48	(45–52)	**	58	(55–61)	58	(55–61)			per unit	0.993	0.0019	–	–	–
Smoker (%)	17	(27%)	10	(24%)		11	(22%)	9	(20%)				–	–	–	–	–
Fitness (VO_2_/kg/FFM)	49.2	(45.8–52.9)	39.7	(36.4–43.2)	***	39.1	(35.9–42.7)	34.7	(31.7–37.9)		###	pf10inc	0.713	0.0888	0.585	(0.379–0.903)	0.0159
WHR	0.91	(0.88–0.94)	0.99	(0.97–1.01)	***	0.96	(0.94–0.98)	1.00	(0.98–1.02)	*	#	pf10inc	4.279	0.012			ns
BMI (kg/m^2^)	25.7	(24.8–26.6)	36.7	(35.6–37.8)	***	26.6	(25.6–27.6)	35.5	(34.4–36.5)	***		pf10inc	3.983	0.0001	3.184	(1.665–6.087)	0.0005
Total fat mass (kg)	20.5	(18.1–22.9)	44.3	(41.4–47.2)	***	22.9	(20.8–25.0)	37.5	(35.3–39.7)	***		per unit	1.008	0.0001	1.007	(1.003–1.011)	0.0018
*metabolic regulation*																	
p-HDL (mM)	1.7	(1.5–1.8)	1.3	(1.2–1.4)	***	1.3	(1.2–1.4)	1.3	(1.2–1.4)			per unit	0.784	0.0001	0.846	(0.740–0.846)	0.0138
p-LDL (mM)	3.6	(3.4–3.9)	3.3	(3.0–3.6)		2.9	(2.6–3.2)	3.0	(2.7–3.3)		###	per unit	0.969	0.2062			ns
p-TAG (mM)	1.0	(0.9–1.2)	1.4	(1.2–1.7)	**	1.6	(1.3–2.0)	1.5	(1.2–1.9)			pf10inc	1.269	0.0063			ns
p-Glucose (mM)	5.1	(5.0–5.2)	5.2	(5.1–5.4)		9.1	(8.2–10.1)	9.0	(8.0–10.1)		###	pf10inc	1.184	0.2514			ns
p-Insulin (pM)	34.8	(29.8–40.6)	68.6	(56.9–82.6)	***	52.6	(43.3–64.0)	101	(82.5–124.0)	***	###	pf10inc	1.278	0.0011			ns
HOMA2-IR	0.66	(0.56–0.76)	1.28	(1.07–1.54)	***	1.22	(1.0–1.50)	2.27	(1.83–2.81)	***	###	pf10inc	1.266	0.0009	–	–	–
HbA1c (%)	5.5	(5.4–5.6)	5.6	(5.5–5.6)		7.3	(6.9–7.8)	6.9	(6.5–7.3)		###	per unit	1.028	0.1086			ns
*Inflammation*																	
p-CRP (mg/l)	1.6	(1.3–1.9)	3.9	(3.1–5.0)	***	2.3	(1.8–3.0)	3.7	(2.9–4.8)	**		pf10inc	1.445	0.0001	1.419	(1.249–1.612)	0.0001
p-TNFα (ng/l)	2.3	(2.2–2.4)	2.6	(2.4–2.8)	**	2.6	(2.4–2.8)	2.9	(2.6–3.1)		##	pf10inc	1.720	0.0212	1.624	(1.015–2.597)	0.0431
p-IL-6 (ng/l)	0.91	(0.78–1.1)	2.1	(1.6–2.7)	***	1.5	(1.2–1.8)	1.9	(1.5–2.3)			pf10inc	1.406	0.0001	1.406	(1.223–1.617)	0.0001
Neutrophils (10^9^/l)	2.91	(2.65–3.19)	3.54	(3.19–3.94)	**	3.76	(3.39–4.16)	3.88	(3.52–4.28)		###	pf10inc	2.748	0.0001	2.702	(2.000–3.649)	0.0001
p-Calprotectin (ng/ml)	58.7	(54.1–63.7)	81.2	(73.1–90.2)	***	68.0	(61.8–74.8)	73.8	(66.7–81.7)				–	–	–	–	–

The study population was divided into 4 groups on the basis of obesity (above or below 30 kg/m^2^) and diagnosis of type 2 diabetes (T2D): Normal glucose tolerance (NGT)/Non-obese, NGT/Obese, T2D/Non-obese and T2D/Obese. Data are presented as numbers for categorical variables and as means or geometric means with 95% confidence interval (CI) for continuous variables. #) Difference between glycemia group (NGT vs. T2D); #) p<0.05, ##) p<0.01, ###) p<0.001. *) Difference between obesity groups within each glycemia group. *) p<0.05, **) p<0.01, ***) p<0.001. For age, BMI, total fat mass, p-HDL, p-TAG, p-IL-6, and p-calprotectin, there was an interaction between glycemia group and obesity. Hence, analyses were stratified for glycemia group and only comparisons within glycemia groups are shown. As calprotectin levels were log-transformed to approximate a normal distribution, regression coefficients (RC) and 95% CIs were back-transformed, hence estimating the factor change of calprotectin level attributable to a 1-unit change in the predictor (per unit) or to a per factor-of-10 increase (pf10inc). The multivariate analysis was adjusted for age, sex, current smoking and HOMA2-IR.

### Plasma calprotectin is a marker of BMI

Univariate- and multivariate regression analyses with plasma calprotectin as the dependent variable are shown in table 1. In univariate regression analysis, plasma calprotectin was positively associated with BMI (Fig. 1C), waist-hip-ratio (WHR), total fat mass, fasting insulin, HOMA2-IR, triglyceride, neutrophils and CRP, and negatively associated with HDL, LDL, IL-6, TNFα, and there was a trend for VO_2max_. In multiple regression analysis (adjusting for age, sex, current smoking and HOMA2-IR), plasma calprotectin was positively associated with BMI, total fat mass, neutrophils, CRP, TNF-α, IL-6, and negatively associated with HDL (table 1). When we further adjusted for VO_2max_, plasma calprotectin was no longer associated with TNF-α (p = 0.053). Plasma calprotectin was associated with BMI after adjustment for CRP (p = 0.042), TNF-α (p = 0.0007), and neutrophils (p = 0.011), but just failed to reach significance after adjustment for IL-6 (p = 0.051).

## Discussion

In the present study we demonstrated that obese subjects have elevated plasma calprotectin compared to non-obese healthy controls. In a multivariate regression analysis adjusted for age, sex, smoking and fasting insulin we found a strong positive correlation of plasma calprotectin with BMI, even when adjusting for CRP and neutrophil number. Even though calprotectin has been found to be expressed in and released from skeletal muscle [2], [6], we found no evidence of a dysregulation of one subunit of calprotectin, S100A8, in obese or type 2 diabetic subjects on the mRNA level, suggesting that the increased levels of calprotectin seen in obese subjects are derived from another source than skeletal muscle or that the level of S100A8 mRNA in skeletal muscle in humans has too much individual variation compared with plasma calprotectin. In support of this, we found no correlation between plasma calprotectin and S100A8 mRNA (data not shown). However, whether or not an increase in S100A8 mRNA is required for an increase in release of calprotectin from skeletal muscle is currently unknown. One may speculate that the increase in plasma IL-6 seen in healthy obese subjects compared to lean subjects may be the driving force behind the increase in plasma calprotectin in healthy obese subjects, as IL-6 is known to upregulate S100A8 and S100A9 mRNA in skeletal muscle [6], although an acute increase in systemic IL-6 levels did not increase plasma calprotectin [6].

Interestingly, serum calprotectin and expression in monocytes have been found to be increased in type 1 diabetes patients and have been suggested to be involved in the pathogenesis of type 1 diabetes[9]. However, in the current study, we found no correlation between plasma calprotectin and parameters of glucose homeostasis in the multivariate regression analysis, suggesting the role of calprotectin in glucose homeostasis regulation is minimal.

Heterodimeric S100A8/S100A9, the form measued in this study, but not the monomeric forms, has been shown to increase detrimental effects caused by advanced end glycation products (AGEs) and may therefore play a role in triggering atherosclerosis in subjects associated with high levels of AGEs[10]. The negative correlation between HDL cholesterol and plasma calprotectin may thus indicate a role for calprotectin in the pathogenesis of atherosclerosis. However, whether or not calprotectin directly contributes to any pathological mechanisms in obese subjects is presently unknown but warrants further investigation.

In conclusion, we found that high plasma calprotectin levels were a marker of obesity independently of classical inflammation markers such as CRP, TNF-α and circulating neutrophil number.
